# Individual gain/loss attitude, conditional cooperation, and random shifting in a public goods game

**DOI:** 10.1186/1471-2202-13-S1-P111

**Published:** 2012-07-16

**Authors:** Dongil Chung, Jaeseung Jeong

**Affiliations:** 1 Virginia Tech Carilion Research Institute, Roanoke, VA, USA; 2 Salem Veteran Affairs Medical Center, Salem, VA, USA; 3 Department of Bio and Brain Engineering, Korea Advanced Institute of Science and Technology (KAIST), Daejeon, South Korea

## 

The public goods game (PGG) is an economic game that is frequently used to measure human cooperation and free-riding behavior. Various empirical studies over two decades have addressed the emergence of cooperation and the gradual propagation of free-riding. However, most of the models in the previous studies did not investigate the interactions between and dynamics of cognitive motivations. The aim of the current study was to investigate the dynamic interaction between the opposing cognitive motivations underlying free-riding and cooperation during repeated trials.

We used a computational model that simulates iterative threshold PGG with binary decision-making under three different conditions. Our model consisted of a cognitive process component that encodes multiple forms of objective information, an affective process component that encodes internal states (fear, greed, and social well-being), and a random shifting component.

The individual loss and gain attitudes of the agent determined the initial and converged free-riding ratios. We observed that conditional cooperation established distinguishable free-riding patterns between conditions and that the random shifting component mimicked the frequent shifting of human behavior. Furthermore, this model could be extended to a schizophrenia-like model by adjusting the integration of loss information and social well-being learning rates.

## Conclusions

We conclude that individual gain/loss attitudes, conditional cooperation, and random shifting, the components included in our model as non-objective information, are required to explain local and global strategic free-riding decision patterns.

